# Safety of human serum albumin infusion in heart failure patients with hypoproteinemia: a propensity score-matched analysis

**DOI:** 10.1016/j.clinsp.2025.100659

**Published:** 2025-04-24

**Authors:** Tongqing Yao, Yinhua Xi, Fei Chen, Hao Lin, Jun Qian, Xuebo Liu

**Affiliations:** a Department of Cardiology, Songjiang Hospital Affiliated to Shanghai Jiao Tong University School of Medicine, China; b Department of Cardiology, Tongji Hospital, School of Medicine, Tongji University, China

**Keywords:** Human serum albumin, Heart failure, Hypoproteinemia, Propensity score-matched analysis

## Abstract

**Objectives:**

Heart failure, especially in the elderly, is a growing global issue. This research explores the safety of human albumin infusion in treating heart failure patients with hypoalbuminemia, despite limited clinical data on its use with diuretic therapy.

**Methods:**

Data were collected from the Medical Information Mart for Intensive Care III (MIMIC-III) database, which included a cohort of 6094 patients diagnosed with heart failure. Propensity score matching, logistic regression, Kaplan-Meier curves, and Locally Weighted Scatterplot Smoothing (LOWESS) curves were used to examine how albumin administration correlates with different clinical results. The evaluated results comprised in-hospital mortality, cumulative fluid output over 24 hours, Length of Stay in the Intensive Care Unit (ICU LOS), and overall hospitalization duration (hospital LOS).

**Results:**

Infusing albumin was found to be markedly linked with higher in-hospital mortality among heart failure patients whose serum albumin levels were ≤ 2.9 g/dL (*p* < 0.001). However, no statistically significant correlation was found between the patients with levels > 2.9 g/dL (*p* = 0.62). Following propensity score matching in the ≤ 2.9 g/dL serum albumin group, albumin infusion was linked to prolonged hospital and ICU stays (*p* = 0.002, *p* < 0.001), but showed no significant association with total 24 hour fluid output and 90-day survival (*p* = 0.173, *p* = 0.656).

**Conclusion:**

The use of albumin has been linked to increased risk-adjusted mortality during hospitalization, as well as prolonged stays in both hospital and ICU settings for patients with heart failure and low levels of serum albumin. Physicians should exercise caution when considering the administration of albumin in these cases.

## Highlights

• Heart failure cohort analysis using the MIMIC-III database of 6094 patients.
• Impact of clinical factors on heart failure prognosis and patient outcomes.
• Exploring biomarkers associated with heart failure progression and mortality.
• Data-driven insights into heart failure management from ICU patient data.
• Predictive modeling of heart failure outcomes based on the MIMIC-III dataset.

## Introduction

Heart Failure (HF) stands as a leading cause of hospitalization and readmission in the elderly population, resulting in significant financial burdens,^1^ and surpassing most cancers in mortality rates.^2^ Managing volume overload in heart failure generally includes approaches like limiting fluid intake and utilizing diuretic therapy. Resistance to diuretics can emerge during the advanced phases of the disease, even with continuous treatment.

Research indicates that the effectiveness of furosemide, a commonly used diuretic in HF, heavily depends on plasma albumin levels. Approximately 95 % of furosemide molecules bind to plasma albumin, forming a complex that targets proximal tubular cells via anion transporters, thereby enhancing urine production.^3^ Serum albumin levels are recognized as a marker of overall health status^4^ and are associated with conditions such as malnutrition, inflammation, and cardiovascular diseases such as HF and coronary artery disease.^5^

Clinicians often face challenges in managing HF patients with furosemide resistance due to low serum albumin levels. In such cases, the administration of human albumin has been considered to counteract hypoalbuminemia and potentially improve diuretic response. However, human albumin is a colloid solution that can exacerbate HF symptoms by increasing the volume load. Its use in HF treatment remains controversial and is typically reserved for well-established indications due to its high cost and limited availability.

To thoroughly investigate the influence of human albumin infusion on mortality among hospitalized heart failure patients with hypoalbuminemia, the authors conducted an extensive retrospective study utilizing data from the Medical Information Mart for Intensive Care-III (MIMIC-III) database. This study seeks to offer insights into the effectiveness and safety of albumin infusion in this particular group of patients. It highlights the importance of careful evaluation, acknowledging its impact on heart failure outcomes despite its suggested benefits in addressing resistance to furosemide.

## Methods

### Design of the study

Data from the Medical Information Mart for Intensive Care III (MIMIC-III) database, which covered the years 2001 to 2012, were used in this retrospective cohort study.^6^ The MIMIC-III database provides high-quality, comprehensive data gathered from Beth Israel Deaconess Medical Center (BIDMC) critical care units. This database has performed well in many model prediction studies and retrospective studies.^7^, ^8^, ^9^, ^10^ Relevant data were extracted from this database (certificate code: 32299459) in accordance with the guidelines set forth in the Presenting of Studies carried out utilizing the Observational Routinely Collected Health Data (RECORD) statement.^11^

### Selection of participants

**Fig. 1:**
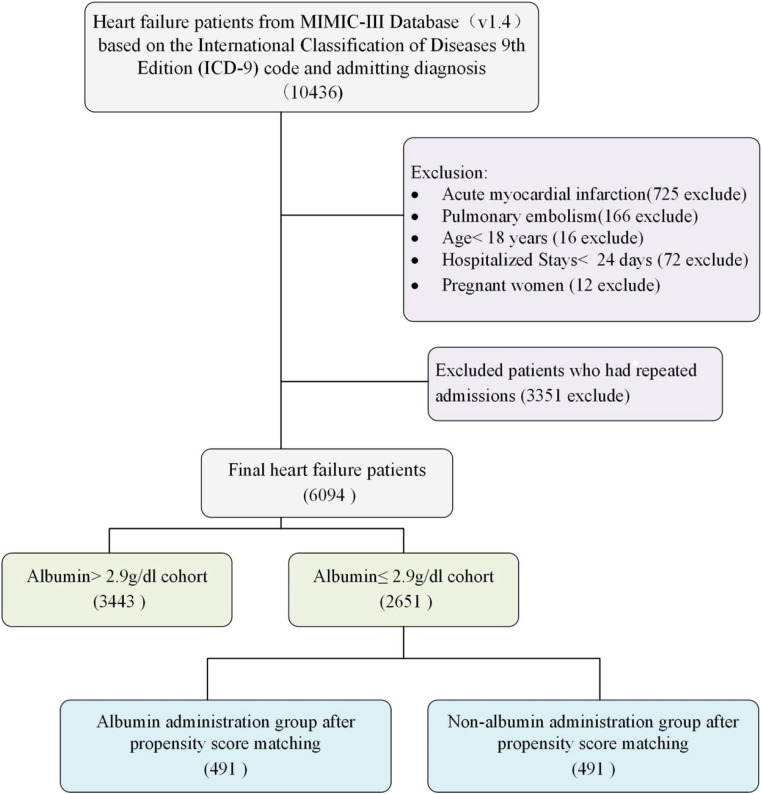
Study flow diagram in the present study.

In the MIMIC-III database, a total of 10,436 patients identified as having Heart Failure (HF) were initially identified through International Classification of Diseases codes and admitting diagnoses. HF was diagnosed according to ICD-9 (42,821, 42,822, 42,823, 42,831, 42,832, 42,833, 42,841, 42,842, and 42,843) and ICD-10 (I5021, I5022, I5023, I5031, I5032, I5033, I5041, I5042, I5043, I50811, I50812, and I50813). To emphasize heart failure independently, excluding complicating factors such as recent heart attacks or sudden pulmonary embolisms, 725 and 166 patients, respectively, were excluded due to their high associated mortality and varying HF severity based on infarct characteristics. Further exclusions included 72 patients hospitalized for less than 24 hours, 16 patients under 18 years-old, and 3363 individuals with repeated admissions or pregnancy. Ultimately, the study included 6094 HF patients (Fig. 1), with only their first ICU stay analyzed when multiple stays occurred.

### Variable extraction

SQL (Structured Query Language) was employed to gather initial patient characteristics upon admission to the Intensive Care Unit (ICU). These encompassed age, gender, and Sequential Organ Failure Assessment (SOFA) scores. Administration of human albumin was duly documented. Essential laboratory parameters like initial albumin levels, White Blood Cell (WBC) count, glucose levels, potassium, platelet count, creatinine, blood urea nitrogen, hematocrit and sodium were also collected. Timestamps for admission and discharge, as well as entry and exit from the ICU, were documented. Pre-existing conditions such as coronary artery disease, diabetes, and Chronic Obstructive Pulmonary Disease (COPD) were recognized using ICD-9 codes.

### Outcomes

The primary focus of the research was the rate of mortality during hospitalization, both with and without the use of albumin. Secondary aspects assessed included the duration of hospitalization, time spent in the ICU, fluid output over a 24-hour period, and survival rates over a 90-day period.

### Statistical analysis

Analysis of the data was carried out using Stata/SE 12.0 and R x64 4.1.3. Continuous variables were reported as means with their corresponding standard deviations or as medians. Categorical variables were demonstrated as percentages and counts. To compare groups, Student's *t*-test was employed for normally distributed continuous variables, whereas Wilcoxon rank-sum or Kruskal-Wallis tests were utilized for non-normally distributed continuous variables. Categorical variables were assessed using either the χ² test or Fisher's exact test based on appropriateness. ROC curves were employed to predict in-hospital mortality at varying serum albumin levels, identifying an optimal threshold value. A multivariate logistic regression, stratified according to a specified cut-off point, assessed the association between hospital mortality and baseline variables identified as clinically relevant or statistically significant in univariate analysis (*p* < 0.05). Propensity Score Matching (PSM) was employed to account for covariates, ensuring the reliability of the outcomes.^12^ One-to-one nearest neighbor matching was conducted with a caliper width of 0.05. The matched cohort was then analyzed to derive outcomes. The relationship between the quantity of albumin infusion and hospital mortality was visualized using the LOWESS smoothing algorithm. All statistical analyses were conducted using a two-tailed approach, with a significance level set at 0.05.

## Results

### Baseline characteristics

A total of 6094 patients underwent serum albumin concentration assessments during their hospital stay. Among these individuals, 4951 recovered, whereas 1143 did not, resulting in a hospital mortality rate of 9.8 %. Table 1 delivers a detailed comparison of the baseline characteristics between survivors and non-survivors. Survivors were notably younger with a mean age of 71.5 years (±13.6) compared to non-survivors, who had an average age of 75.1 years (±12.8) (*p* < 0.001). The non-survivors had significantly greater Sequential Organ Failure Assessment (SOFA) scores (6 [4‒9] vs. 4 [3‒7], *p* < 0.001) and lower initial serum albumin levels (2.8 [2.3‒3.2] vs. 3.1 [2.7‒3.6] g/dL, *p* < 0.001). The proportions of survivors using furosemide and metoprolol were 64.4 % and 60.2 %, respectively, while the corresponding proportions for non-survivors were 66.1 % and 55.5 %. Apart from differences in sex, maximal potassium levels, and Chronic Obstructive Pulmonary Disease (COPD), all other baseline elements showed statistically substantial disparities among non-survivors and survivors.

**Table 1:** Baseline characteristics of demographics between survivors and non-survivors.

**Variables**	**Survivors** **(*n* = 4951)**	**Non-survivors** **(*n* = 1143)**	***p***
Age (years)	71.5 ± 13.6	75.1 ± 12.8	< 0.001
Men, *n* (%)	2641 (53.3)	614 (53.7)	0.053
SOFA score	4(3‒7)	6 (4‒9)	< 0.001
ALB_infu, *n* (%)	694 (14.0)	246 (21.5)	< 0.001
**Laboratory data**			
WBC_max (10×9/L)	14.5 (10.9‒19.5)	17.9 (12.3‒25.1)	< 0.001
PLT_max (10×9/L)	328.5 ± 160.5	285.7 ± 156.3	< 0.001
Potassium_max (mEq/L)	5.5 ± 1.1	5.6 ± 1.0	0.07
Sodium_max (mEq/L)	143.2 ± 4.6	144.4 ± 5.8	< 0.001
Cre_max (mg/dL)	1.4 (1.0‒2.3)	2.1 (1.3‒3.5)	< 0.001
BUN_max (mg/dL)	107 (36‒173)	122 (57‒183)	< 0.001
Hematocrit ( %)	31.3 ± 5.1	30.8 ± 4.9	0.006
ALB_C (g/dL)	3.1 (2.7‒3.6)	2.8 (2.3‒3.2)	< 0.001
**Comorbidities**			
Diabetes	2023 (40.9)	406 (35.5)	0.001
COPD, *n* (%)	290 (5.9)	57 (5.0)	0.252
Coronary disease, *n* (%)	2272 (49.5)	351 (30.7)	< 0.001
**Pharmacological background**			
Furosemide, *n* (%)	3189 (64.4)	456 (66.1)	0.165
Metoprolol, *n* (%)	2976 (60.2)	634 (55.5)	0.056

ALB_infu, Albumin infusion; ALB_C initial, Albumin concentration; SOFA, Sequential Organ Failure Assessment; WBC_max, maximum White Blood Cell count during hospital stay; PLT_max, maximum Blood Platelet count during hospital stay; Hematocrit, initial Hematocrit during hospital stay; Potassium_max, maximum Potassium during hospital stay; Cre_max, maximum Creatinine during hospital stay; BUN_max, maximum urea Nitrogen during hospital stay; Sodium_max, maximum Sodium during hospital stay; COPD, Chronic Obstructive Pulmonary Disease.

### Analysis of mortality by logistic regression

**Fig. 2:**
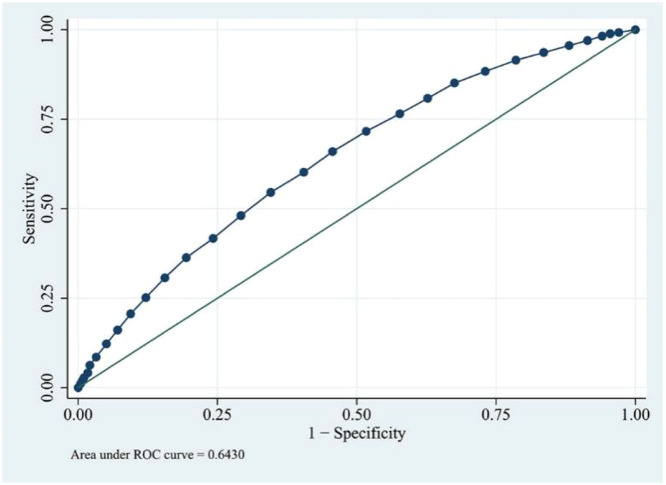
Performance in predicting in-hospital mortality using albumin concentration. Prediction performance was based on ROC. The area under curve was 0.643. The cut-off value was 2.9 g/dL. ROC, Receiver Operator Curves.

**Fig. 3:**
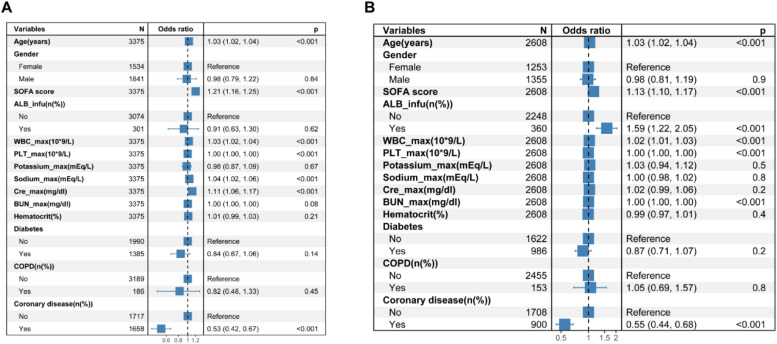
Effects of variables on hospital mortality in stepwise multivariate logistic regression analysis in heart failure patients with albumin > 2.9 g/dL (A) and with albumin ≤ 2.9 g/dL (B). ALB_infu, Albumin Infusion; SOFA Sequential Organ Failure Assessment; WBC_max, maximum White Blood Cell count during hospital stay; PLT_max, maximum blood Platelet count during hospital stay; Hematocrit, Initial hematocrit during hospital stay; Potassium_max, maximum Potassium during hospital stay; Cre_max, maximum Creatinine during hospital stay; BUN_max, maximum Urea Nitrogen during hospital stay; Sodium_max, maximum Sodium during hospital stay; COPD, Chronic Obstructive Pulmonary Disease.

Based on the cutoff value determined from the ROC curves (Fig. 2), the cohort was split into two groups: those with albumin levels > 2.9 g/dL and those with ≤ 2.9 g/dL. Logistic regression was utilized to forecast in-hospital mortality among these cohorts. Stepwise logistic regression was applied to ascertain noteworthy factors, employing a significance level set at p 〈 0.05. In patients with serum albumin levels 〉 2.9 g/dL, the administration of albumin did not result in a statistically significant difference in-hospital mortality (*p* = 0.62) (Fig. 3A). Conversely, in patients with serum albumin ≤ 2.9 g/dL, albumin administration was found to be linked with increased mortality, as indicated by stepwise logistic regression analyses (OR = 1.59, *p* < 0.001) (Fig. 3B).

### Primary and secondary effects following PSM

**Fig. 4:**
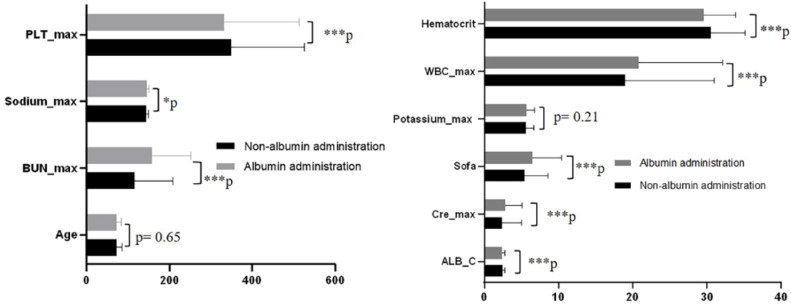
Characteristics of covariate between albumin administration and non-albumin administration with albumin ≤ 2.9 g/dL (*p < 0.05; ***p < 0.001). ALB_infu, Albumin infusion; Sofa, Sequential Organ Failure Assessment; WBC_max, maximum White Blood Cell count during hospital stay; PLT_max, maximum Blood Platelet count during hospital stay; Hematocrit, Initial hematocrit during hospital stay; Potassium_max, maximum Potassium during hospital stay; Cre_max, maximum Creatinine during hospital stay; BUN_max, maximum Urea Nitrogen during hospital stay; Sodium_max, maximum Sodium during hospital stay; ALB_C, initial Albumin concentration.

**Fig. 5:**
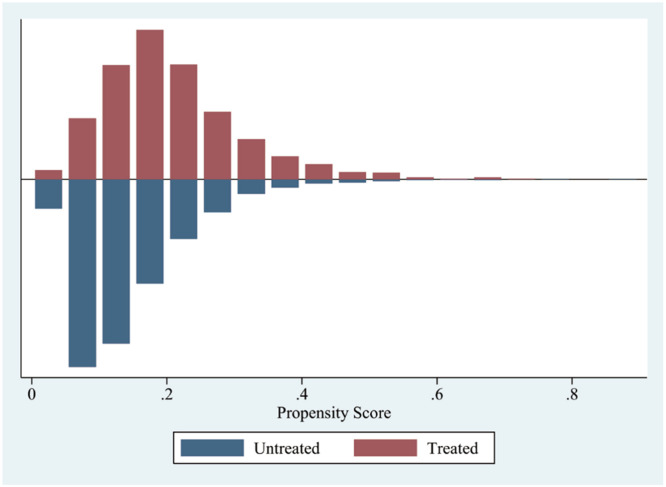
Covariates including initial albumin concentration, SOFA score, maximum white blood cell count, maximum blood platelet count, maximum creatinine level, maximum serum urea nitrogen level, maximum serum sodium level, chronic obstructive pulmonary disease was adjusted by Propensity Score Matching (PSM) between albumin administration and non-albumin administration groups with albumin ≤ 2.9 g/dL and the caliper was 0.05. After PSM, 982 patients with albumin ≤ 2.9 g/dL were stayed.

Mortality rates showed significant variation among patients whose serum albumin levels were ≤ 2.9 g/dL, as noted earlier. Further examination focused on the baseline characteristics of the groups that received albumin infusion and those that did not within this subset. Except for potassium levels and age, all other baseline factors, including maximum white blood cell count, SOFA score, initial albumin concentration, maximum platelets count, initial hematocrit, maximum creatinine, maximum urea nitrogen, and maximum sodium, exhibited notable distinctions between the groups (see Fig. 4). Additionally, there was a notable difference in the prevalence of COPD (Table 2). To minimize discrepancies in covariates between these groups, Propensity Score Matching (PSM) was employed (Fig. 5, Table 3).

**Table 2:** Comorbidities characteristics between albumin administration and non-albumin administration groups with albumin ≤ 2.9 g/dL.

**Comorbidities**	**Albumin administration**	**Non-albumin administration**	**p-value**
Diabetes, *n* (%)	130 (35.9)	880 (38.4)	0.356
COPD, *n* (%)	10 (2.8)	147 (6.4)	0.006
Coronary disease, *n* (%)	138 (38.1)	784 (34.3)	0.151

COPD, Chronic Obstructive Pulmonary Disease.

**Table 3:** Covariate were adjusted by Propensity Score Matching (PSM) between albumin administration and non-albumin administration groups with albumin ≤ 2.9 g/dL.

Variable	Unmatched	Mean		%reduct		p-value
	Matched	Treated	Control	%bias	|bias|	
ALB_C	U	2.4102	2.4841	−20.5		< 0.001
	M	2.4102	2.3929	4.8	76.6	0.226
WBC_max	U	20.819	19.013	11.9		< 0.001
	M	20.819	21.018	−1.3	89.0	0.133
PLT_max	U	332.95	349.73	−9.3		< 0.001
	M	332.95	344.87	−6.6	29.0	0.165
Cre_max	U	2.7627	2.3613	8.2		< 0.001
	M	2.7627	2.7723	−0.2	97.6	0.639
BUN_max	U	158.52	116.48	45.1		< 0.001
	M	158.52	155.57	3.2	93.0	0.553
Sodium_max	U	144.75	144.23	9.7		0.04
	M	144.75	144.53	4.1	58.1	0.552
Hematocrit	U	29.582	30.539	−21.2		< 0.001
	M	29.582	28.984	13.2	37.6	0.075
SOFA	U	6.5377	5.4341	31.2		< 0.001
	M	6.5377	6.3605	5.0	83.9	0.82
COPD	U	0.02851	0.06566	−17.6		0.006
	M	0.02851	0.03055	−1.0	94.5	0.805

ALB_C, initial Albumin Concentration; SOFA, Sequential Organ Failure Assessment; WBC_max, maximum White Blood Cell count during hospital stay; PLT_max, maximum blood Platelet count during hospital stay; Hematocrit, initial Hematocrit during hospital stay; Cre_max, maximum Creatinine during hospital stay; BUN_max, maximum urea nitrogen during hospital stay; Sodium_max, maximum Sodium during hospital stay; COPD, Chronic Obstructive Pulmonary Disease.

**Fig. 6:**
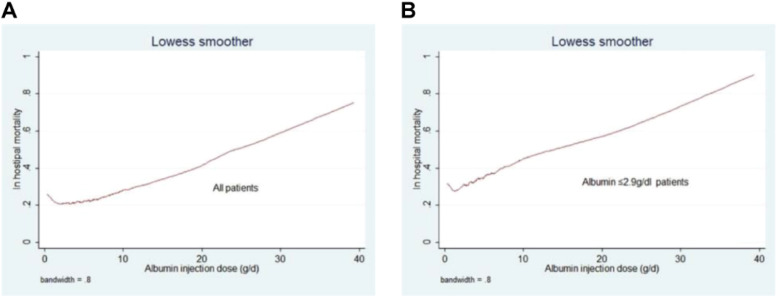
The relationship between amount of human albumin infusion and in-hospital mortality in the HF group (A) and HF plus albumin ≤ 2.9 g/dL group (B).

Following Propensity Score Matching (PSM), the authors analyzed primary and secondary outcomes in 982 patients whose serum albumin levels were ≤ 2.9 g/dL. The group receiving albumin showed elevated rates of in-hospital mortality and longer hospital and ICU stays (in-hospital mortality: *p* = 0.001; hospital stay: *p* = 0.002; ICU stay: *p* < 0.001). However, the administration of albumin infusion did not demonstrate a significant correlation with the total fluid output within the 24-hour period (*p* = 0.173). Furthermore, the authors examined the relationship between the quantity of human albumin infused within 24 hours and in-hospital mortality rates in both the overall Heart Failure (HF) cohort and the subgroup with HF plus albumin ≤ 2.9 g/dL. Increased albumin infusion was significantly linked to elevated hospital mortality rates in both groups (Fig. 6).

**Fig. 7:**
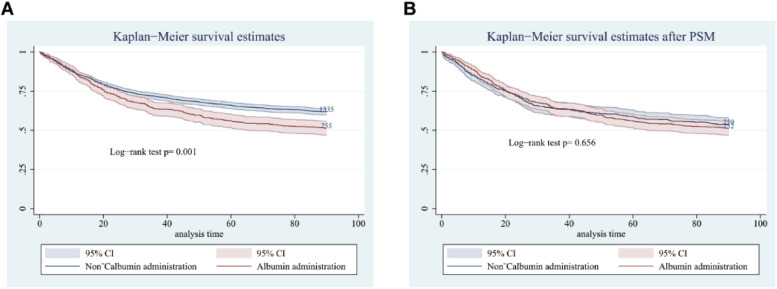
Survival during 90 days in albumin administration and non-albumin administration subgroups before (A) and after (B) PSM when albumin ≤ 2.9 g/dL. PSM, Propensity Score Matching.

Kaplan-Meier curve analysis comparing 90-day survival between patients who received albumin and those who did not, among those with serum albumin ≤ 2.9 g/dL, revealed a significant association with decreased 90-day survival before PSM (*p* = 0.001) (Fig. 7A), but no significant association after PSM (*p* = 0.656) (Fig. 7B).

## Discussion

The present study found that the use of human albumin was connected with increased risk-adjusted mortality during hospitalization, as well as prolonged hospital and ICU stays, in patients diagnosed with Heart Failure (HF) and serum albumin levels ≤ 2.9 g/dL, compared to individuals who did not receive albumin. This finding is particularly relevant when considering the role of congestion in heart failure, as albumin is intricately related to the congestion status, which is one of the most intriguing parameters in this context.

Albumin, produced by the liver, serves several vital functions in the body. It contributes significantly to the colloid oncotic pressure of plasma, which helps maintain the integrity of capillary membranes and fluid balance across these membranes.^5^ Previous research has shown that albumin also has anticoagulant and antiplatelet properties.^13^, ^14^, ^15^, ^16^ Furthermore, it exerts anti-inflammatory effects by inhibiting proinflammatory mediators.^17^ Albumin serves as an anti-inflammatory agent by specifically diminishing cytokine-triggered endothelial activation, which is pivotal in conditions such as atherosclerosis.^18^ Serum albumin levels are influenced by factors affecting its synthesis, breakdown, and distribution within and outside vascular compartments. Since 1989, the concentration of serum albumin has been recognized as a reliable prognostic indicator for Cardiovascular Diseases (CVD).^19^

In line with these findings, Massari F et al. highlighted the significance of congestion in heart failure patients and how it influences treatment outcomes.^20^ They emphasized the importance of monitoring and managing congestion as a key factor in the treatment of heart failure patients. Furthermore, in their earlier study, Massari F et al. discussed the role of congestion in Heart Failure with preserved Ejection Fraction (HFpEF), noting that effective management of congestion is crucial for improving patient outcomes.^21^

HF is a complex clinical condition involving cardiac and circulatory dysfunction, disruptions in neurohormonal and biochemical balance, and impaired organ function. In patients with HF, albumin production is often reduced due to conditions such as liver congestion, congestive cirrhosis or intestinal edema. Hypoalbuminemia is prevalent in HF patients and is an independent predictor of adverse results and poorer survival.^22^ Furthermore, reduced levels of albumin have been linked to an increased prevalence of HFpEF.^23^^,^^24^ The present research investigated the correlation between initial serum albumin levels and in-hospital mortality through the utilization of ROC curves. In line with previous studies, the research indicates that low levels of albumin predict the likelihood of death from any cause in patients hospitalized for heart failure, identifying an optimal threshold at 2.9 g/dL. Given the critical importance of serum albumin, there is considerable interest in exploring the potential benefits of administering albumin to patients suffering from hypoproteinemia. For instance, in critically ill patients, albumin infusion has been suggested to enhance organ function.^25^ Caraceni et al.^26^ conducted a study demonstrating a significant improvement in 18-month survival rates among patients with decompensated cirrhosis who received albumin infusion. However, a recent randomized controlled trial concluded that there is no additional benefit observed by aiming for albumin levels of 30 g/L or higher in hospitalized patients experiencing decompensated cirrhosis.^27^ Notably, this analysis of MIMIC-III data revealed that albumin administration was associated with increased mortality in heart failure patients with albumin levels ≤ 2.9 g/dL, as determined through stepwise multivariate logistic regression analyses.

To reduce variations in baseline characteristics among patients who received albumin and those who did not, the authors employed Propensity Score Matching (PSM). Covariates showing initial imbalances included albumin infusion, baseline albumin levels, SOFA score, peak creatinine, peak white blood cell count, peak platelets count, baseline hematocrit, peak potassium, peak urea nitrogen, peak sodium, and coronary artery disease. The present research revealed that the use of albumin was linked to increased in-hospital mortality rates, and extended hospital and ICU stays compared to patients not receiving albumin, particularly among heart failure patients with hypoalbuminemia. Additionally, the authors examined the impact of albumin volume infused within 24-hours on hospital mortality, revealing that higher volumes correlated with increased rates of mortality during hospitalization.

Volume overload often presents a challenge in managing patients with Heart Failure (HF), typically addressed through fluid restriction and diuretic use. However, resistance to diuretics remains a significant issue. One key reason for this resistance is the reduced delivery of diuretic agents to their intended site.^28^ Albumin serves as a carrier for drugs, facilitating their transport to target organs.^3^^,^^29^ When albumin levels are low (hypoalbuminemia), the secretion of diuretic agents into the tubular lumen can be diminished.^30^^,^^31^ Clinical trials have shown that administering intravenous furosemide complexed with albumin can rapidly enhance urine production in patients who have low protein levels and are unresponsive to diuretic therapy.^3^ For patients with conditions like acute respiratory distress syndrome or acute lung injury and hypoalbuminemia, combining furosemide with albumin therapy has shown significant benefits, including improved oxygenation, achieving a net negative fluid balance, and enhancing hemodynamic stability compared to using furosemide alone.^32^ Additionally, research by Chalasani et al. indicated similar levels of urinary sodium excretion and furosemide excretion between cirrhotic patients receiving furosemide alone and those receiving furosemide combined with albumin therapy for ascites.^33^

Currently, there is no clear evidence supporting the advantages of administering albumin for heart failure. Nonetheless, certain doctors administer albumin infusions to address resistance to furosemide in heart failure patients with low albumin levels. In the present research, after accounting for other influencing factors, the authors found no significant correlation between albumin infusion and 24-hour urine output. Furthermore, albumin, being a colloid, increases fluid volume, which could potentially exacerbate symptoms of heart failure.

The present research is the first to explore the utilization of albumin therapy in hospitalized HF patients with low albumin levels, revealing no significant clinical benefits. On the contrary, these findings suggest that administering albumin to these patients is linked to higher in-hospital mortality rates and longer stays in both hospital wards and ICUs. These outcomes can be interpreted in multiple ways. Initially, as a colloid, albumin can increase fluid volume, potentially exacerbating adverse outcomes in HF. Although albumin might alleviate diuretic resistance and promote urine production, the resulting increase in fluid volume may counteract these potential benefits. Secondly, the physiological roles of albumin in inflammation suppression and its anticoagulant and antiplatelet effects may require more time to manifest. The present study's duration was limited to a maximum of 90-days, during which the authors observed no reduction in 90-day mortality following albumin infusion. Longer-term follow-up studies could uncover benefits from extended albumin treatments. Lastly, modifications of albumin, such as reversibly oxidized albumin (HNA1) and irreversibly oxidized albumin (HNA2),^34^ should be considered. Evaluating the HNA1 to HNA2 ratio and the conversion of HNA1 into human mercaptalbumin could provide deeper insights.^35^

The present study also highlighted that patients with coronary disease showed lower in-hospital mortality rates, possibly because clinicians closely monitored medications and adhered to restrictions on fluid volume and infusion rates in these cases. Moreover, excluding patients with myocardial infarction may have resulted in a cohort with less severe coronary artery disease.

These findings reflect the practical clinical implications of albumin administration in HF patients with low albumin levels, indicating no significant benefits. Therefore, the results do not support the routine use of albumin therapy in this patient population. Nonetheless, clinicians should not dismiss albumin therapy entirely but should instead carefully consider its appropriate application.

## Limitations

This study encountered several limitations. Initially, the dataset extracted from MIMIC-III for N-terminal pro-brain-type natriuretic peptide comprised about 2000 entries, but due to a high incidence of missing data, this variable had to be excluded from analysis. Consequently, the definition of heart failure relied solely on the International Classification of Diseases, 9th Edition, and admitting diagnoses. Moreover, cardiac function grades were unavailable from this database.

Secondly, this research was a retrospective cohort study conducted at a solitary institution utilizing the MIMIC-III database, encompassing the period from 2001 to 2015. Consequently, the results are likely to predominantly mirror medical practices specific to that era and location.

Thirdly, several variables, diuretic usage, including 24-hour input amount and body mass index, had missing data exceeding 50 % and were therefore excluded from analysis. Other unmeasured variables, such as differences in hemodynamic monitoring techniques (e.g., central venous pressure measurement, transthoracic echocardiography), may have influenced the outcomes but were not accounted for.

Fourthly, adjusting for various factors influencing initial central venous pressure levels posed challenges in conducting a retrospective observational study. Factors included the infusion rate of albumin and other fluid solutions. Moreover, the study did not delve into the potential influence of protein concentration on the outcomes, which presents an avenue for future investigation.

Finally, the study did not thoroughly examine the causal link between the administration of human albumin and mortality rates. The longer length of stay observed in the heart failure cohort might not be directly related to albumin administration, as the length of stay is influenced by various complex clinical factors. The retrospective study design limits the ability to establish causality and may lead to selection bias and information bias. The authors acknowledge that these conclusions may be limited by the limitations of this study design and may not apply to the most recent treatments for heart failure. Therefore, the authors suggest that future studies should have a prospective design in order to better understand the efficacy and safety of human serum albumin in patients with heart failure. Future randomized studies comparing the effects of human and non-human albumin administration are necessary to provide further insights.

## Conclusion

In this research, the study found that giving albumin to patients with heart failure who have low albumin levels was linked to higher adjusted mortality rates during hospitalization and within 28-days, along with prolonged stays in both the hospital and intensive care unit. Interestingly, albumin infusion did not show any association with fluid output within 24-hours or mortality rates after 90-days. These findings suggest that doctors should be careful when thinking about giving albumin to heart failure patients who have low levels of protein in their blood.

## CRediT authorship contribution statement

**Tongqing Yao:** Conceptualization, Writing – review & editing. **Yinhua Xi:** Software. **Fei Chen:** Software. **Hao Lin:** Data curation. **Jun Qian:** Data curation. **Xuebo Liu:** Conceptualization, Funding acquisition, Writing – review & editing.

## Declaration of competing interest

The authors declare no conflicts of interest.
